# Gold‐FISH enables targeted NanoSIMS analysis of plant‐associated bacteria

**DOI:** 10.1111/nph.19112

**Published:** 2023-06-28

**Authors:** Hannes Schmidt, Stefan Gorka, David Seki, Arno Schintlmeister, Dagmar Woebken

**Affiliations:** ^1^ Centre for Microbiology and Environmental Systems Science University of Vienna Vienna 1030 Austria; ^2^ Doctoral School in Microbiology and Environmental Science University of Vienna Vienna 1030 Austria; ^3^ Large‐Instrument Facility for Environmental and Isotope Mass Spectrometry, Centre for Microbiology and Environmental Systems Science University of Vienna Vienna 1030 Austria

**Keywords:** biological nitrogen fixation, *in situ* hybridization, microbial activity, NanoSIMS, plant growth‐promoting bacteria, plant–microbe interaction, rhizosphere bacteria

## Abstract

- Bacteria colonize plant roots and engage in reciprocal interactions with their hosts. However, the contribution of individual taxa or groups of bacteria to plant nutrition and fitness is not well characterized due to a lack of *in situ* evidence of bacterial activity.
- To address this knowledge gap, we developed an analytical approach that combines the identification and localization of individual bacteria on root surfaces via gold‐based *in situ* hybridization with correlative NanoSIMS imaging of incorporated stable isotopes, indicative of metabolic activity.
- We incubated *Kosakonia* strain DS‐1‐associated, gnotobiotically grown rice plants with ^15^N–N_2_ gas to detect *in situ* N_2_ fixation activity. Bacterial cells along the rhizoplane showed heterogeneous patterns of ^15^N enrichment, ranging from the natural isotope abundance levels up to 12.07 at% ^15^N (average and median of 3.36 and 2.85 at% ^15^N, respectively, *n* = 697 cells).
- The presented correlative optical and chemical imaging analysis is applicable to a broad range of studies investigating plant–microbe interactions. For example, it enables verification of the *in situ* metabolic activity of host‐associated commercialized strains or plant growth‐promoting bacteria, thereby disentangling their role in plant nutrition. Such data facilitate the design of plant–microbe combinations for improvement of crop management.

## Introduction

Plants are tightly associated with a high number and diversity of microorganisms. They colonize all plant organs both endophytically and epiphytically, with roots representing a major interface of microbe–microbe and plant–microbe interactions (Andrews & Harris, 2000; Vandenkoornhuyse *et al*., 2015). Microbial activity is thought to play a central role in plant fitness, for example, through stress mitigation and protection from plant pathogens (Shalev *et al*., 2022; Trivedi *et al*., 2022) and also in plant nutrition, for example, by providing plant growth‐limiting nutrients such as phosphorous or nitrogen (Hirota *et al*., 1978; Jiang *et al*., 2021).

Symbiotic associations are often confined to specific organs (e.g. root nodules in legumes), which facilitates the investigation of bidirectional flow of substrates and signals between plant and microbes due to a general understanding of the site of interaction. Microorganisms not confined to specific organs in and on plants, however, may also interact with their host, but knowledge on their site of action and their contribution to plant fitness and nutrition is scarce. For example, soil bacteria that fix atmospheric dinitrogen (N_2_; i.e. free‐living diazotrophs) are known to colonize roots of globally important crops such as rice, sugarcane, and maize without forming a symbiotic association *sensu stricto*. They have been suggested to provide fixed N to their host plant in numerous studies (Boddey *et al*., 1995; Van Deynze *et al*., 2018; Pang *et al*., 2023). Diazotrophic bacteria were even commercialized for farming, yet (1) their ^15^N_2_ fixation activity, (2) their sites of action along and within plant organs, and (3) the transfer of fixed N to host plants has been a matter of debate due to missing *in situ* evidence (James, 2000; Cassán & Diaz‐Zorita, 2016; Chalk *et al*., 2017; Dixon & Hartmann, 2017).

Localizing microorganisms on and within plant organs indicates potential sites of microbial activity and represents the first milestone in investigating the interaction between plants and their microbiome *in situ*. To localize plant‐associated microorganisms of interest, fluorescence microscopy can be combined with fluorescence *in situ* hybridization (FISH). FISH allows to mark the microbe of interest with fluorophores and has been successfully applied to localize bacteria on plant roots or leaves and to study their spatial distribution (Watt *et al*., 2006; Peredo & Simmons, 2018; Martin *et al*., 2020). For root surfaces, bacteria have been mainly observed in grooves between plant cells walls while their abundances vary significantly from root tips to mature regions, which may indicate preferential sites of plant–microbe interaction (Bloemberg *et al*., 2000; Watt *et al*., 2006; Schmidt & Eickhorst, 2014).

The second milestone is to assess the activity of microorganisms *in situ*, which can be achieved by tracing the incorporation of stable isotopes such as ^13^C or ^15^N into microbial biomass (Pett‐Ridge & Firestone, 2017; Angel *et al*., 2018). Combined with nanoscale secondary ion mass spectrometry (NanoSIMS), it allows to visualize the enrichment of these stable isotope tracers at the microbial scale and thus to measure the activity of single microbial cells within a given population (Behrens *et al*., 2008; Musat *et al*., 2008; McGlynn *et al*., 2015). NanoSIMS of microbes of unknown identity has been successfully applied to investigate assimilatory processes at a microscale in root and soil ecosystems (Herrmann *et al*., 2007; Clode *et al*., 2009; Nuccio *et al*., 2013; Eichorst *et al*., 2015; Worrich *et al*., 2017; Gorka *et al*., 2019; Witzgall *et al*., 2021). However, the targeted analysis of specific microorganisms via previous identification is largely missing.

NanoSIMS approaches often employ imaging of (1) microbes removed from their environment (thereby loosing spatial context) or (2) impregnated cross‐sections of roots (thereby limiting the number of analyzed microbial cells). The latter was performed in a recent study combining FISH with ^15^N‐targeted NanoSIMS imaging of an endophytic marine bacterium in cross‐sections of resin‐impregnated seagrass roots, which represents a breakthrough in identifying target microorganisms within roots and assessing their activity *in situ* (Mohr *et al*., 2021). In order to analyze epiphytic root‐colonizing bacteria, however, resin impregnation does not work well due to the high topography of root surfaces, which leads to only few bacterial cells in the focal plane after sectioning the sample longitudinally. Root topography also limits the possibility to correlate fluorescence imaging (roots immersed in liquid medium) with NanoSIMS (dry roots required) as the location of target organisms cannot be retrieved after drying of the root.

Here, we present a novel approach that allows targeted detection of microbial cells that actively incorporated stable isotopes on root surface by combining gold‐based 16S rRNA *in situ* hybridization, scanning electron microscopy (SEM), and NanoSIMS. This method combines the identification and localization of single microbial cells across different imaging scales within their topographically pronounced plant–soil microenvironment comprising high‐resolution chemical imaging via NanoSIMS. As a proof of principle, we performed ^15^N–N_2_ gas incubations of rice plants associated with a rhizosphere‐isolated bacterium capable of colonizing rice roots and fixing N_2_ *in situ* under gnotobiotic conditions. Our approach is widely applicable across the field of plant–microorganism–soil interactions and has the potential to clarify current uncertainties regarding microbial *in situ* activity.

## Materials and Methods

### Cultivation of *Kosakonia* strain DS‐1 and gnotobiotic experiment with rice

*Kosakonia* strain DS‐1 (GenBank: CP040677.1) was grown under hypoxic conditions (1% oxygen) at 19°C without shaking in semisolid NFCC medium (Mirza & Rodrigues, 2012). The medium contained either no N source (natural isotope abundance control) or 5 mM ^15^N–NH_4_Cl (99%, Lot#: I1‐128750; Cambridge Isotope Laboratories, Andover, MA, USA) as the sole N source (utilized for determination of the isotope label dilution effect introduced by Gold‐FISH). Cells were harvested at mid‐log phase by centrifugation and fixed in 1 × PBS – formaldehyde (4% final concentration) for 3 h at 4°C, washed twice in 1 × PBS, and stored in 1 × PBS : EtOH (v/v) at −20°C.

Gnotobiotic experiments with young rice plants and *Kosakonia* strain DS‐1 were conducted as reported previously (Schmidt *et al*., 2018). Briefly, rice seeds (cv IR64) were surface‐sterilized in 5% NaOCl and germinated on sterile LB medium for 10 d at 25°C. Sterilized glass tubes were filled with a mixture of gellan gum medium (1.5%; Gelrite, Carl Roth, Germany) and N‐free Yoshida solution (without NH_4_NO_3_; Yoshida *et al*., 1976). Cells of *Kosakonia* strain DS‐1 were cultivated under N‐fixing conditions in semisolid NFCC medium without any N source as described above. Cells were harvested at mid‐log phase by centrifugation and re‐suspended in N‐free Yoshida solution to a density of *c*. 10^4^ cells μl^−1^. The radicle of 10‐d‐old rice seedlings that were devoid of bacterial and fungal contamination, as visually inspected by eye, were submerged in 500 μl of *Kosakonia* strain DS‐1 inoculum for 30 min under sterile conditions. Individual inoculated seedlings were transplanted into glass tubes with the radicle facing downwards into the medium. Yoshida solution was added on top of the medium surface (*c*. 1 cm) to simulate the submerged conditions of wetland rice cultivation. Autoclaved wool was used to seal the tubes, which were placed for 3 wk in a glasshouse with a day‐and‐night cycle of 14 and 10 h accompanied by average temperatures of 30°C and 22°C, respectively. Every 3 d, the tubes were opened under sterile conditions to replace Yoshida solution, to inspect for potential contamination, and to allow for a complete exchange of the atmosphere within the tube. Sterile controls without inoculum were prepared accordingly and did not show bacterial colonization as detailed in Schmidt *et al*. (2018).

### ^15^N–N_2_ incubations of young rice plants and associated bacteria

Tubes with 3‐wk‐old plants were opened under sterile conditions. Excess Yoshida solution was replaced with autoclaved H_2_O_MQ_ to minimize the possibility that microorganisms reside in the liquid on the surface. The tubes were sealed with autoclaved rubber stoppers and flushed with Helium for 2 min. Afterward, 38.5 ml of helium were withdrawn with a syringe and substituted by O_2_ (99.9 at% ^16^O, Alphagaz 1; Air Liquide, Düsseldorf, Germany), ^15^N–N_2_ (98 at%, batch: MBBB0968V; Campro Scientific, Berlin, Germany), and CO_2_ (99.9 at% ^12^C, N45; Air Liquide) to reach final volume fractions of 21%, 39.5%, and 0.2%, respectively. Enclosed tubes were incubated for 72 h in the glasshouse. Afterward, each tube was opened under sterile conditions. A subset of the roots was weighed and subjected to chemical fixation in 2.5% formaldehyde for 3 h at 4°C, washed in 1 × PBS twice, and stored in 1 × PBS : EtOH (40 : 60, v/v) at −20°C. The remaining roots, stems, and leaves were dried at 60°C for 48 h. These samples were processed by milling (MM400; Retsch, Haan, Germany; 2 × 1 min, 30 m s^−1^) and measured via an Elemental Analyzer (EA 1110; CE Instruments, Milan, Italy) coupled to a Finnigan MAT Delta Plus IRMS (Thermo Fisher Scientific, Waltham, MA, USA). Control incubations with ^14^N_2_ gas were performed accordingly.

To evaluate the potential contamination of ^15^N–N_2_ gas with ^15^N ammonia (Dabundo *et al*., 2014), the ^15^N–N_2_ gas was tested for ^15^N‐labeled ammonia gas contaminations using the hypobromite oxidation method (Warembourg, 1993; Preisler *et al*., 2007). These measurements indicated the presence of 146 nmol ^15^N ammonia per mol ^15^N–N_2_ on average, which in our case represented 44% of the total ^15^N measured per plant (root and shoot). In turn, this means that 56% of the total amount of ^15^N incorporated per plant originated from N_2_ fixation by root‐associated *Kosakonia* strain DS‐1 (Supporting Information Table S1).

### Gold‐FISH of root‐associated *Kosakonia* strain DS‐1 cells

Gold‐FISH was performed according to Schmidt *et al*. (2012) with slight modifications. A detailed description of the procedure is given in the Supporting Information (Methods S1). Briefly, *in situ* hybridization of bacteria was performed on intact root sections using oligonucleotide probes (EUBI‐III; Biomers.net, Ulm, Germany; Daims *et al*., 1999). For CARD amplification of streptavidin‐binding sites, a biotinylated tyramide solution containing 2% 3‐iodophenolboronic acid (Sigma‐Aldrich) was used. Afterward, gold nanoparticles and fluorophores were bound to biotin by incubation in AlexaFluor®488FluoroNanogold™‐Streptavidin (Nanoprobes, Yaphank, NY, USA). To enhance the grain size of gold nanoparticles for SEM and NanoSIMS, each root segment was immersed in a gold developer solution (GoldEnhance™ EM Plus; Nanoprobes).

### Preparation and microscopic characterization of samples before NanoSIMS analysis

All samples were deposited on antimony‐doped silicon wafer platelets (7.1 × 7.1 × 0.75 mm, Active Business Company, Marquartstein, Germany) precoated with Vectabond (Vector Laboratories Inc., Newark, CA, USA) to improve adhesion of specimen to the wafer surface. Suspensions of fixed pure cultures of *Kosakonia* strain DS‐1 were diluted in H_2_O_MQ,_ and 5 μl was spotted onto individual wafers. A confocal laser scanning microscope (TCS SP8X; Leica Microsystems, Wetzlar, Germany) was used to verify successful Gold‐FISH hybridization on pure cultures and root segments. Silicon wafer platelets with air‐dried root segments were imaged by a SEM (IT 300, JEOL, Freising, Germany) to screen root surfaces for microbial colonization through the detection of gold signals. Regions that showed bacterial colonization, as visualized through gold deposition, were then selected as potential analysis areas for NanoSIMS based on the observed bacterial cell density and root topography. To retrieve appropriate analysis areas by NanoSIMS, the silicon wafer platelets were marked using a laser microdissection microscope (Leica Microsystems). A detailed description of sample preparation including microscopic characterization before NanoSIMS analysis can be found in the Supporting Information (Methods S2).

### NanoSIMS analyses

NanoSIMS measurements were performed on a NanoSIMS 50 l (Cameca, Gennevilliers, France) at the Large‐Instrument Facility for Advanced Isotope Research at the University of Vienna. In order to minimize degradation of the mass resolving power (MRP) due to topography, samples were mounted with a preferentially horizontal alignment of roots inside the NanoSIMS analysis chamber (Gorka *et al*., 2019). Before the data acquisition, analysis areas were preconditioned *in situ* by rastering of a high‐intensity, defocused Cs^+^ ion beam in the following sequence of high and extreme low ion impact energies (HE/16 keV and EXLIE/50 eV, respectively): HE at 25 pA beam current to a Cs^+^ fluence of 5.0E14 ions cm^−2^; EXLIE at 400 pA beam current to a fluence of 5.0E16 ions cm^−2^; and HE at 25 pA to a fluence of 5.0E14 ions cm^−2^. Data were acquired as multilayer image stacks by repeated scanning of a finely focused Cs^+^ primary ion beam (*c*. 80 nm probe size at 2 pA beam current) over areas between 34 × 34 and 72 × 72 μm^2^ at 512 × 512‐pixel image resolution and a primary ion beam dwell time of 5 ms pixel^−1^. Owing to the limited length of the magnet and the physical width of the detectors, both being part of the multicollector mass spectrometer of the NS50L, simultaneous determination of the N isotope composition, and relative gold content was not feasible. Accordingly, each acquisition cycle was split into two half‐cycles conducted at distinct magnetic field strength. In the first half‐cycle, performed at 2673 Gauss, ^12^C^−^, ^12^C^14^N^−^, and ^197^Au^−^ secondary ions were detected. Instantaneously before acquisition of the second half‐cycle, the magnetic field strength was enhanced to 2724 Gauss, enabling parallel detection of ^12^C_2_^−^, ^12^C^15^N^−^, ^31^P^−^, and ^197^Au^−^ secondary ions. ^197^Au^−^ intensity distribution images, designated as ^197^Au^−^(1) and ^197^Au^−^(2), as well as secondary electron images were recorded in both half‐cycles to facilitate drift correction and for monitoring morphological variations originating from continuous sample erosion. We note that, although the filaments were coated by an electrically conducting carbon thin film, a slight build‐up of (positive) charges was yet observed within the analysis areas which we compensated by application of an additional voltage of −20 V on the sample (‘EOW‐offset’). The magnitude of charging remained constant throughout the entire measurements, conducted as long runs with a total acquisition time of up to 39 h. After each completed acquisition cycle, secondary ion beam drift was corrected by automatic beam centering (‘SIBC’) and coaxial lens (‘EOS’) voltage optimization utilizing ^12^C_2_^−^ as reference signal. In addition, the automatic peak centering routine (‘APC’) was applied on each of the recorded secondary ion species except for ^12^C^15^N^−^ and ^31^P^−^, which were, owing to their weak signal intensities, aligned relative to the stronger ^12^C_2_^−^ signal (recorded in the identical half‐cycle). The mass spectrometer was tuned for achieving a mass resolving power (MRP) of > 7000 both for detection of ^12^C^14^N^−^ and ^197^Au^−^ ions.

### Data analysis

NanoSIMS images were generated and analyzed with the OpenMIMS plugin (Poczatek *et al*., 2012) in the image processing package Fiji (Schindelin *et al*., 2012). All images were auto‐tracked for compensation of primary ion beam and/or sample stage drift, and secondary ion signal intensities were corrected for detector dead‐time and quasi‐simultaneous arrival (QSA) of secondary ions, utilizing sensitivity factors (‘beta’ values) of 1.1, 1.06, and 1.05 for C^−^, C_2_^−^, and CN^−^ ions, respectively. Overlays for visual comparison between NanoSIMS and backscattered electron (BSE) SEM images were created in the GNU Image Manipulation Program (v.2.10.12). Images of pure cultures were auto‐tracked and regions of interest (ROIs) were defined in the ^12^C^−^‐ion image (unlabeled and ^15^N‐labeled cells) or the ^12^C^14^N^−^‐ion image (^15^N‐labeled cells with Gold‐FISH), where each ROI corresponded to an individual cell. Attention was paid to exclusively select the bacterial cell lumen. For root surfaces, auto‐tracking was performed utilizing one of the two ^197^Au^−^ signals recorded in each half‐cycle (i.e. ^197^Au^−^(1) or ^197^Au^−^(2)). Slight discrepancies in *xy*‐positioning between half‐cycles were observed, and images were additionally manually aligned based on both ^197^Au^−^ signals to obtain a reasonable match. It should be noted that secondary ion signal intensities generally depend, in addition to the concentration of the analyte, on multiple parameters (Williams, 1992). The primary ion impact angle on the sample surface and the secondary ion emission angle are among these, leading to signal intensity variations related to the surface structure within the analysis area (Thomen *et al*., 2014). In determination of isotope fractions, topography effects are largely canceled since both detected secondary ion species (here, ^12^C^14^N^−^ and ^12^C^15^N^−^) are affected similarly. Nevertheless, mass fractionation in the SIMS measurement process, designated as instrumental mass fraction (IMF), is detectable in high‐sensitivity measurements but can be minimized by proper tuning of the instrument and a sufficiently conservative definition of isotope enrichment in the analysis of isotopically labeled sample material. For analysis of relative elemental concentrations, a proper reference signal needs to be chosen. Here, we used ^12^C^−^ for normalization of the ^197^Au^−^ and ^31^P^−^ signals based on carbon as a matrix element and the similar kinetic energy and angular distribution of atomic secondary ions. Areas exhibiting extremely low signal intensities for ^12^C^−^ and secondary electrons, both being related to topography, were excluded from ROI analysis.

The objective of this image evaluation approach was to characterize gold‐labeled cells. Hence, all ROIs were initially defined based on the ^197^Au^−^ : ^12^C^−^ intensity ratio. Simultaneously, the ^31^P^−^ : ^12^C^−^ intensity ratio was inspected as an indicator for cellular biomass. When ^197^Au^−^ : ^12^C^−^ and ^31^P^−^ : ^12^C^−^ values correlated, ROIs were defined based on both signals. ROIs were selected to interrogate individual cells, or at most small clusters of cells when a visual distinction was not possible. Large clusters that potentially represented bacterial biofilms were omitted. In our workflow, ROIs were initially defined in the early stage of a measurement (Cs^+^ fluence up to 3.7E16 ions cm^−2^), as this particularly refers to the surface‐near regions of the sample, most likely including bacterial cells, especially their gold‐coated surface. However, owing to the topographical complexity of the sample, we also inspected ^197^Au^−^ : ^12^C^−^ hotspots appearing in the later cycles of the measurement.

Regions of interest were analyzed for their ^15^N content by calculating the average value across acquisition cycles per *Analysis Area* (AA1: 1–95, AA2: 20–40, AA3: 2–4), referred to by the ^15^N/(^14^N + ^15^N) isotope fraction designated as at% ^15^N, which was calculated from the ^12^CN^−^ signal intensities via

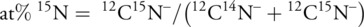




The natural abundance of ^15^N in cellular biomass was inferred from analysis of single cells from *Kosakonia* strain DS‐1 pure cultures (isotopically unlabeled, without Gold‐FISH), yielding 0.362 ± 0.045 at% (mean ± 1 SD). The analytical uncertainty in the ROI‐specific ^15^N content values emerging from the random error in single ion counting was estimated based on Poisson statistics and calculated from the signal intensities (given in total counts within each individual ROI) as:

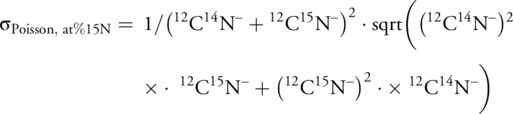




Regions of interest were considered as significantly enriched in ^15^N if the respective at% ^15^N value was (1) higher than the mean plus six standard deviations of the unlabeled control and (2) the counting error (6σ_Poisson_) was smaller than the deviation of the ROI value from the mean of the unlabeled control. We note that each ROI value that fulfilled criterion (1) also passed criterion (2). Accordingly, 0.63 at% ^15^N was defined as a threshold value for classification as significant ^15^N enrichment and applied as the lower bound of the at% ^15^N scale in the isotope distribution images.

The at% ^15^N values from each ROI per *Analysis Area* were plotted in ‘R’ (v.3.6.1; R Development Core Team, 2019) with the package ‘ggplot2’ (Wickham, 2016). Statistically significant differences in the ^15^N content between ROIs selected in different *Analysis Areas* and the natural isotope abundance control were evaluated via ANOVA and Tukey *post hoc* test using the package ‘vegan’ (Oksanen *et al*., 2016).

## Results

### Identification of gold‐labeled bacteria on root surfaces via SEM and NanoSIMS

**Fig. 1:**
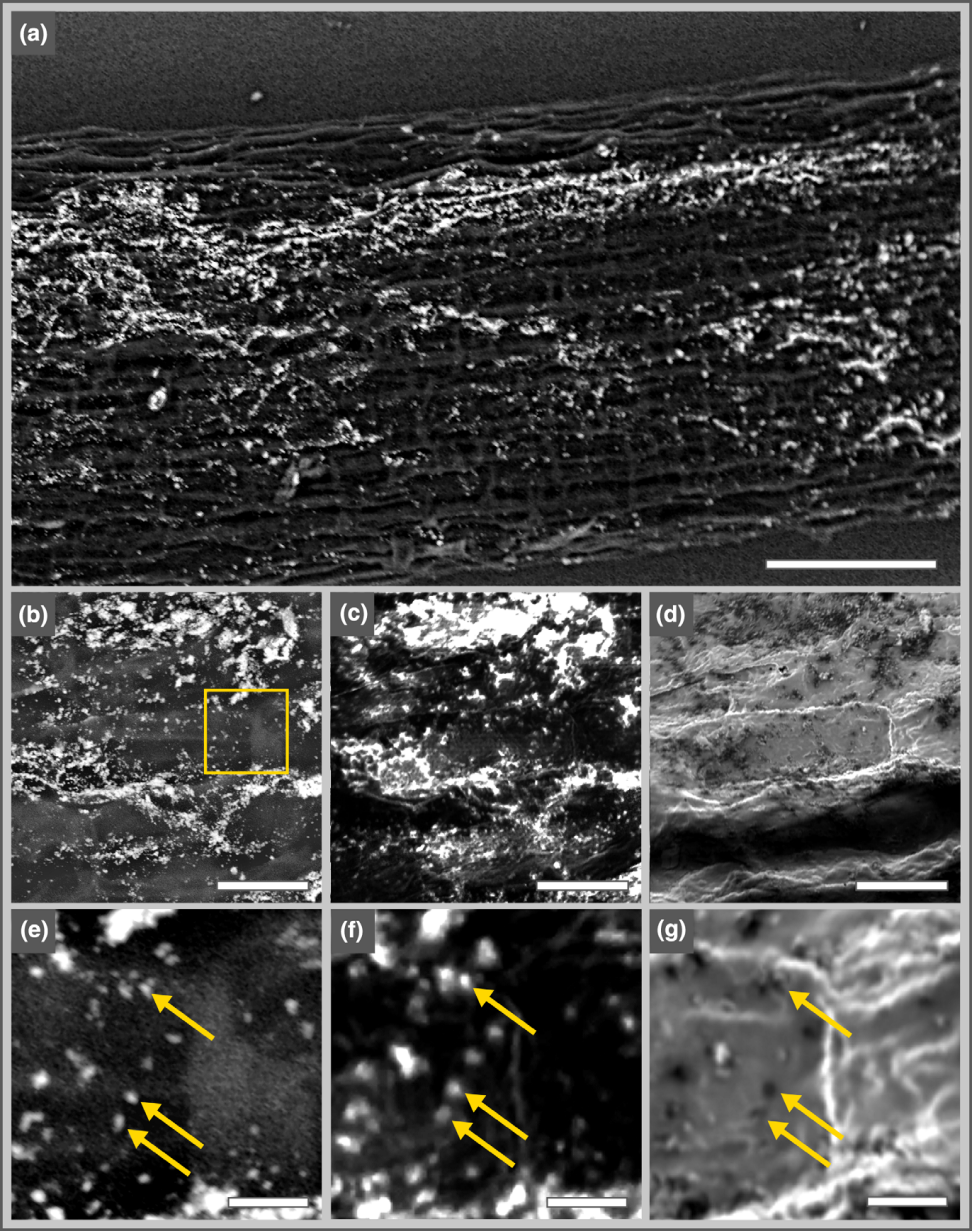
Correlative imaging of gold particles on the root surface via scanning electron microscopy–backscattered electron detection (SEM‐BSE) and nanoscale secondary ion mass spectrometry (NanoSIMS). Gold signals appear as bright spots in SEM‐BSE (a, b, e) and NanoSIMS ^12^C^−^ normalized ^197^Au^−^ secondary ion maps (c, f) while appearing as dark spots in NanoSIMS secondary electron images (d, g). Gold‐colored squares represent the region of the zoom‐in areas displayed in panels (b–d, e–g), respectively. Arrows highlight individual gold particles correlatively detected by the applied imaging techniques. Bars: (a) 100 μm; (b–d) 20 μm; (e–g) 5 μm.

In this study, root segments colonized by *Kosakonia* strain DS‐1 were subjected to our novel Gold‐FISH‐NanoSIMS analytical approach. We employed SEM to select root surface areas according to the criteria of (1) providing a high bacterial colonization density while (2) showing a sufficiently high number of individual bacterial cells and (3) representing a region with modest surface topography to maximize the efficiency and throughput of NanoSIMS analysis. All root segments imaged via SEM showed a similar distribution of gold particles (bright signals, Fig. 1a) along the rhizoplane as commonly observed for root‐colonizing bacteria and *Kosakonia* strain DS‐1 via fluorescence microscopy (Schmidt & Eickhorst, 2014; Schmidt *et al*., 2018). Target cells appeared as individual cells but also in cell clusters on these root surfaces. Potential *Analysis Areas* (AA) were selected according to the criteria mentioned above and correlative gold imaging was performed. Gold particles appeared as bright spots in SEM–BSE images (Fig. 1b,e) and NanoSIMS ^197^Au^−^ secondary ion signal intensity distribution images (Fig. 1c,f), while they were visible as dark spots when imaged via NanoSIMS secondary electron detection (Fig. 1d,g). Images of high magnification of the analyzed AA clearly show that a correlative imaging of gold particles in SEM and NanoSIMS was achieved (Fig. 1e,f). As visible in panels (b,c), gold detection by the NanoSIMS was more sensitive than via SEM, which is consistent with the high yield of Au^−^ secondary ions achieved by Cs^+^ primary ion bombardment (Storms *et al*., 1977). The NanoSIMS secondary electron images revealed that the labeled cells were located on the surface of the root, allowing for a detailed representation of the topography including individual gold‐labeled bacterial cells (Fig. 1d).

### Correlative Au and ^15^N isotope single‐cell imaging on root surfaces

**Fig. 2:**
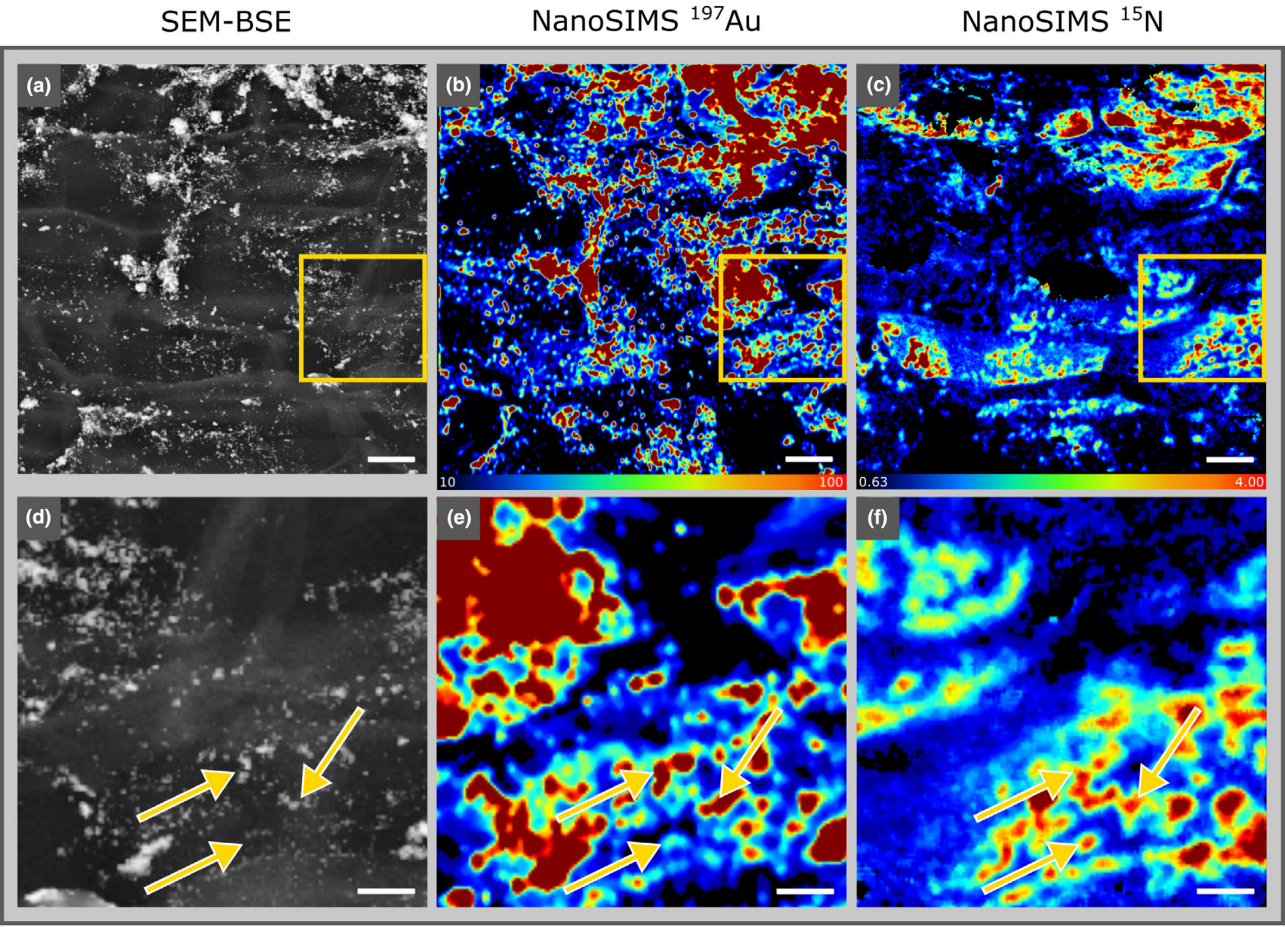
Correlation between gold‐labeled bacteria and regions of ^15^N enrichment close to the root surface. Gold particles appear as bright spots in scanning electron microscopy‐backscattered electron detection (SEM‐BSE) images (a, d) and in warm colors (yellow–red) in nanoscale secondary ion mass spectrometry (NanoSIMS) ^197^Au^−^ secondary ion maps (b, e; image acquisition cycle 5). NanoSIMS‐^15^N content distribution images (c, f; as indicated in the image recorded in acquisition cycle 30). The minimum of the at% ^15^N false‐color scale was set to 0.63, representing the mean + 6 SD of the single‐cell values determined on the unlabeled control. As such, colored areas refer to regions of local ^15^N enrichment (specified in the Materials and Methods section). Gold‐colored squares represent the zoom‐in areas shown in panels (d–f). Arrows highlight individual gold particles correlating with ^15^N enrichment. Bars: (a–c) 5 μm; (d–f) 2 μm.

Another region (AA1) on the same root segment was analyzed to verify whether (1) it was possible to obtain signals for Au and ^15^N enrichment and (2) the distribution patterns would correlate spatially. Achieving spatial correlation is a prerequisite for our analytical approach. Here, as a proof of concept, we sought to link regions of ^15^N isotope enrichment to the presence of gold‐labeled single cells of the diazotroph *Kosakonia* strain DS‐1. In the measurement on AA1, we recorded a total of 110 complete NanoSIMS imaging cycles, each representing an individual map of the elemental gold and ^15^N isotope tracer distribution. The acquisition of such a high number of cycles was performed to cover most of the microbial cells distributed over the root surface exhibiting a considerable topography. Comparable to the previous acquisition (Fig. 1), there was a nearly perfect correlation between the gold signal intensity pattern observed by SEM‐BSE and the NanoSIMS ^197^Au^−^ secondary ion map (Fig. 2d,e). In addition, these gold signals were found to correlate with areas/spots of ^15^N enrichment, suggesting that individual bacterial cells were both labeled with Au and enriched in ^15^N. In terms of NanoSIMS analysis, it is worth noting that the presence of Au was permanently indicated in earlier image acquisition cycles than the enrichment in ^15^N, which was confirmed in measurements of single cells from pure cultures of *Kosakonia* strain DS‐1 (Fig. S1). This effect may be explained by the localization of gold as being deposited preferentially in and on the cell wall and membranes of bacterial cells via Gold‐FISH (Schmidt *et al*., 2012) and/or the inherently high sputter yield of gold (Chambers & Fine, 1992).

### Stepwise definition of single‐cell ROIs

Our analytical approach employs the identification of bacterial cells located on the root surface through the detection of gold particles by SEM. A necessary step to render the gold nanoparticles (1.4 nm) visible for SEM is their enhancement in size via auto‐metallography. Although the protocol developed by the supplier for enhancement of gold nanoparticles generates only low levels of background gold signals through a reduced auto‐nucleation, one might still observe nonspecific deposition in environmental samples, which could lead to the inclusion of false‐positive signals (Eickhorst & Schmidt, 2015). More importantly, the structural complexity of the given root specimen could lead to a shift of co‐occurring signals over several acquisition cycles. Therefore, we used a stepwise approach to identify single‐cell ROIs for analysis of ^15^N enrichment in *Kosakonia* strain DS‐1, based on the concurrent detection of Au, P, and ^15^N. As described in the Materials and Methods section, single‐cell ROIs were selected only for individual cells and small clusters of cells, while large aggregates potentially representing bacterial biofilms were omitted. ROIs were first defined based on the ^12^C^−^ normalized ^197^Au^−^ signal intensity which resulted in a total of 406 ROIs for AA1. Within these ROIs, we then searched for the presence of phosphorus via the ^12^C^−^ normalized ^31^P^−^‐signal intensity and the ^15^N isotope enrichment in consecutive acquisition cycles (see Fig. S2). We categorized these into 364 ROIs identified by the ^197^Au^−^ : ^12^C^−^ and P^−^ : ^12^C^−^ intensity ratios, of which 269 ROIs also exhibited an enrichment in ^15^N.

### Quantitative image analysis reveals ^15^N enrichment of single bacterial cells

**Fig. 3:**
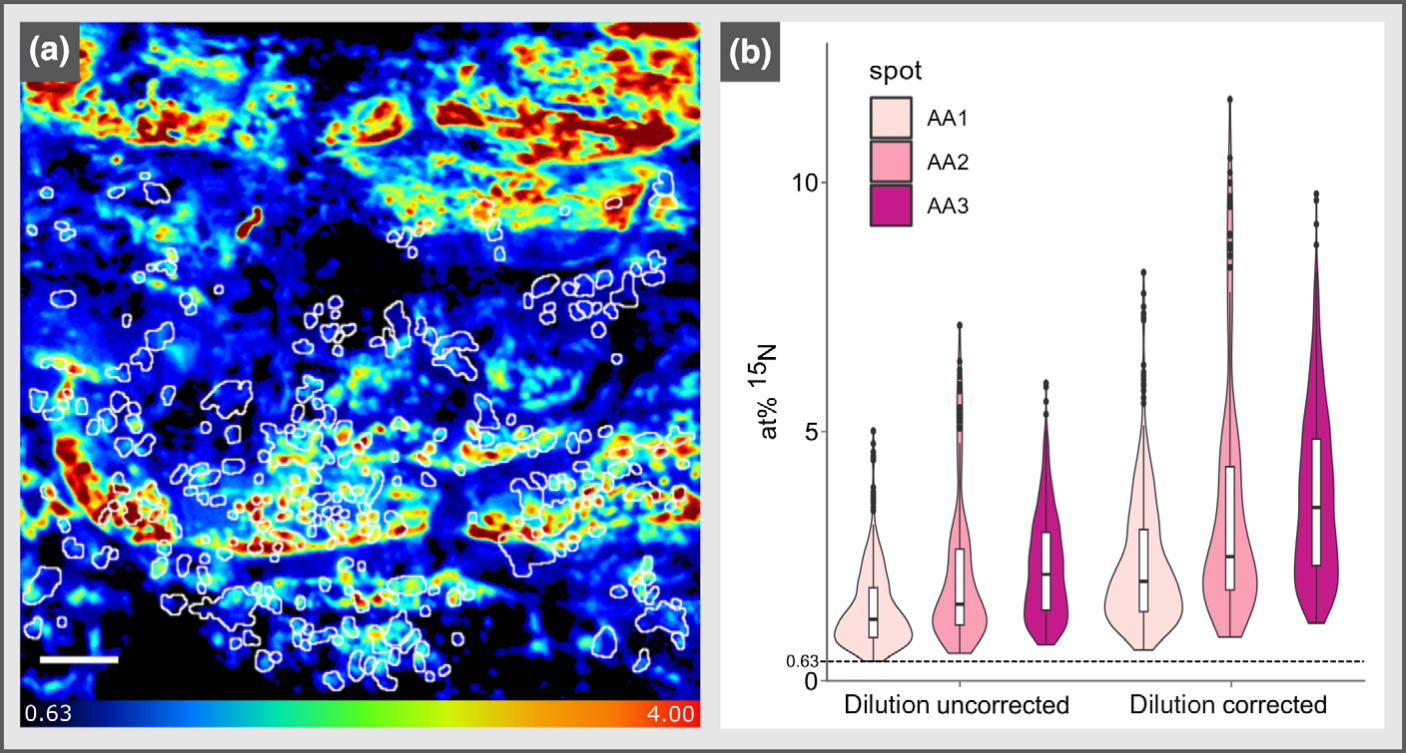
Regions of interest (ROI) and quantification of ^15^N enrichment. (a) Nanoscale secondary ion mass spectrometry (NanoSIMS) at% ^15^N image including selected ROIs (white circular shapes) with varying ^15^N enrichment (warm colors, yellow–red) or values close to ^15^N natural abundance (dark blue) in *Analysis Area* 1 (AA1). Note that the displayed image refers to the sum of all cycles of the multicycle acquisition. A feature of this visualization is that the ^15^N enrichment is not recognizable within all ROIs as it appears only in a few cycles of the acquisition (see also Supporting Information Fig. S4; Video S1). Details on ROI selection can be found in section ‘Stepwise definition of single‐cell ROIs’. Bar, 5 μm. (b) Violin plot showing summary statistics of the ^15^N content determined as means within selected ROIs across acquisition cycles of three distinct *Analysis Areas* (AA1–AA3) as compared to the natural isotopic abundance measured in single cells from pure cultures of *Kosakonia* strain DS‐1 (see the Materials and Methods section for details). Boxes within violins represent the first and third quartiles while the solid line indicates the median. Values are shown either as raw data or corrected for ^15^N isotopic label decrease caused by the Gold‐fluorescence in situ hybridization (FISH) treatment (see Fig. S3). All ROIs showed significant levels of isotopic enrichment (*P* < 0.0001; specified in the Materials and Methods section).

This subset of ROIs (*n* = 269) was then used for the assessment of ^15^N enrichment of single bacterial cells in AA1 (Fig. 3a). ROIs showed a tracer content up to 4.82 at% ^15^N with an average and median of 1.77 and 1.59 at% ^15^N, respectively. ROIs in AA2 (*n* = 170) and AA3 (*n* = 258) showed an average/median of 2.16/1.60 and 2.23/2.02 at% ^15^N, respectively (Fig. 3b) with individual ROIs yielding values of up to 7.36 at% ^15^N in AA2 and 6.08 at% ^15^N in AA3. The average tracer content of all individual ROIs measured in the three AAs (*n* = 697) was 2.05 at% ^15^N, which is well above the selected threshold value of 0.63 at% ^15^N for indication of significant isotope enrichment (see the Materials and Methods section for the details in definition). A common feature of FISH applications is the decrease in isotopic label contents due to loss of cellular biomass through permeabilization and the deposition of compounds with natural isotopic abundance (Musat *et al*., 2014; Woebken *et al*., 2015; Stryhanyuk *et al*., 2018; Meyer *et al*., 2021). In this study, we detected an average reduction of ^15^N enrichment by 39% per individual bacterial cell due to Gold‐FISH treatment (Fig. S3), which is within the range of ^15^N label dilution observed for CARD‐FISH treatments (recently reviewed and extended by Meyer *et al*., 2021). Consequently, we corrected the values quantified via image analysis by application of a dilution factor of 1.63, leading to a robust estimation of the actual *in situ* ^15^N content of single cells of *Kosakonia* strain DS‐1 on the rhizoplane in this experiment (Fig. 3b). For AA1‐3, these results showed that the average ^15^N content was 2.90, 3.54, and 3.65 at% ^15^N before Gold‐FISH, respectively, with the highest values of > 10 at% ^15^N for individual ROIs.

## Discussion

We present a novel approach for targeted investigation of the *in situ* activity of single bacterial cells in the root–soil interface, which represents a key analytical step towards a better understanding of plant–microorganism interactions (Pett‐Ridge & Firestone, 2017). We tested our analytical approach on root samples of rice plants that were grown in a N‐free medium but associated with the N_2_‐fixing strain *Kosakonia* strain DS‐1. To our knowledge, this study represents the first report that combines phylogenetic identification and localization of individual bacteria at the rhizoplane within their microhabitat via stable isotope incubation and nanoscale isotopic imaging.

### Technical considerations of the presented analytical approach

Conventional FISH techniques hamper a direct correlation of target microorganisms localized on root surfaces with NanoSIMS imaging. Fluorescence imaging for localizing single microbial cells along root surfaces (i.e. longitudinal sections) requires mounting of root specimens under ‘immersed’ conditions with mounting medium to provide a high optical resolution while preventing rapid bleaching of fluorescent dyes. By contrast, NanoSIMS imaging operates under ultra‐high vacuum, which makes dry samples a necessary requirement. Drying of roots, however, changes their morphology and makes a correlation of ‘immersed’ fluorescence images with ‘dry’ NanoSIMS images practically impossible. Moreover, individual bacteria detected by FISH and fluorescence microscopy can hardly be correlated to cell‐like structures observed through SEM at the same location. The application of Gold‐FISH, however, makes use of elemental tagging that facilitates the precise localization of microbial target cells in a dry state and at a resolution compatible with NanoSIMS (see Figs 1, 2, S5), representing two major advantages of the presented analytical approach. By labeling individual bacteria with gold nanoparticles, it allows identifying select regions of interest (i.e. with high density of target cells) and subsequent chemical imaging of this region, for example, specific stable isotopes of interest.

For downstream analysis of NanoSIMS data of *Kosakonia* strain DS‐1 cells colonizing the root surface of rice, we first selected ROIs representing single bacterial cells based on specific gold signals. These ROIs were then screened for the presence of phosphorus, which is assumed to be proportionally higher within the bacterial cell as compared to the bacterial environment (Sterner & Elser, 2002). We selected ROIs that showed both Au‐ and P‐associated signals and only those ROIs that additionally showed ^15^N enrichment were selected for quantitative analysis. However, after analyzing the data we observed that those ROIs containing both Au and P were the least enriched in ^15^N, especially when compared to the Au‐only ROIs (Fig. S6). This did not interfere with our results but suggests that including P as selection criterion of ROIs might have excluded some cells that showed both the presence of Au and ^15^N enrichment, and that P may not be a necessary additional indicator for microbial cells in our workflow.

On average, 74% of all ROIs containing Au + P detected on the root surface also showed an isotope label content above 0.63 at% ^15^N (AA1). Hence, not every bacterial cell identified via Au + P showed ^15^N enrichment indicating that not every *Kosakonia* strain DS‐1 cell was actively taking up ^15^N in our experiment. In turn, not every ^15^N enriched region showed a clear Au signal, suggesting that the Gold‐FISH‐labeling did not provide detection of each individual cell that actively incorporated the isotopic tracer on the root surface. Insufficient cell wall permeabilization and/or inaccessibility of the probe binding site can be a reason why FISH‐based approaches can fail to detect all target cells in an environment (Amann & Fuchs, 2008). The former may play a role in Gold‐FISH, which contains a CARD reaction, an additional streptavidin‐biotin step, as well as auto‐metallographic enhancement of gold nanoparticles. The CARD reaction included in our protocol enables an increased deposition of additional gold on gold nanoparticles which overcomes low signal‐to‐noise ratios reported for standard Gold‐ISH (Kubota *et al*., 2014) and thus represents an essential component of the presented workflow. It is possible that nanoparticles are sometimes not accessible for the reaction to take place inside the cell. However, applications of Gold‐FISH to mixed bacterial cultures showed highly specific labeling of all target cells with gold on polycarbonate filters (Fig. S7; Notes S1). In addition, even if not all target cells were detected on the rhizoplane in the three regions analyzed in our study, the high number of ROIs representing individual bacterial cells enriched in ^15^N captured in all AAs (*n* = 697) was more than enough to perform downstream quantitative analyses.

### *In situ* N_2_ fixation of *Kosakonia* strain DS‐1 associated with rice roots

In this study, we found evidence for *in situ* N_2_ fixation of *Kosakonia* strain DS‐1 colonizing the root surface of wetland rice variety IR64. While *c*. 44% of the ^15^N tracer found in aboveground and belowground plant tissue could have resulted from ^15^N ammonia present in ^15^N–N_2_ gas used for incubation (see Notes S2; Table S1), at least 56% of the ^15^N enrichment must have been derived from biological nitrogen fixation through *Kosakonia* strain DS‐1 in our gnotobiotic system. Among those bacteria, we observed a varying ^15^N enrichment of individual cells on the surface of rice roots. This suggests that some cells of *Kosakonia* strain DS‐1 had more actively taken up ^15^N than others of the same community. This is in line with previous NanoSIMS studies, which revealed heterogeneous physiological activities among members of even clonal bacterial populations (Volland *et al*., 2018; Calabrese *et al*., 2019). While the ^15^N enrichment of individual cells within each analysis area varied strongly, the average/median at% ^15^N values across the three analysis areas did not. This indicates the absence of site‐specific ^15^N‐fixation activities of *Kosakonia* strain DS‐1 cells at three different spots along the root. Recently, members of the bacterial genus *Kosakonia* were reported to be associated with a variety of crop plants and suggested to provide plant growth‐promoting services, potentially also through biological nitrogen fixation (Brock *et al*., 2013; Gu *et al*., 2014; Becker *et al*., 2018). In our gnotobiotic system, *Kosakonia* strain DS‐1 was found to actively fix N_2_ on rice roots 3 wk after inoculation of rice seedlings. A substantial part of ^15^N fixed by the bacteria was detected in the plant biomass via bulk IRMS measurements, which indicates that *Kosakonia* strain DS‐1 has the potential to support plant growth with N under N‐limiting conditions. This notion is supported by the broadening of the ^15^N distribution pattern with increasing NanoSIMS erosion depth (Fig. S4), which could indicate a local ^15^N enrichment of plant material in close spatial proximity to N‐fixing bacteria. These observations are preliminary and need to be carefully interpreted due to the above‐mentioned levels of ^15^N ammonia in our experiment. Supporting our observations with root‐associated ^15^N fixation activity in field experiments would render *Kosakonia* strain DS‐1 an interesting candidate as potential inoculant that could help alleviate N fertilizer use in a variety of cropping systems.

### Gold‐FISH NanoSIMS is highly complementary to existing approaches analyzing microbial activity

We believe that our analytical approach will complement the existing array of tools to assess microbial activity in environmental samples (for an overview see Hatzenpichler *et al*., 2020). While meta‐omics and cultivation‐based approaches can provide first evidence which members of a community are potentially involved in a specific process, visualizing substrate uptake by single cells can provide direct confirmation of their activity (Mohr *et al*., 2021; Wasmund *et al*., 2021). Maintaining the spatial arrangement is especially important for root‐associated epiphytic bacteria, whose heterogeneous colonization patterns are correlated to morphological features of the root surface (Watt *et al*., 2006; Schmidt *et al*., 2018). We previously reported that almost no root‐associated cells were lost after numerous washing and hybridization steps in a similar CARD‐FISH protocol, indicating that the spatial organization of bacterial colonization remained intact in the presented approach (Schmidt & Eickhorst, 2014). Spatial attributes need to be conserved and analytically resolved in case we want to understand and verify potential interactions, as well as their frequency and strength, between bacterial groups and plants. For example, a recent study identified a surprisingly high proportion of potential diazotrophs in bacterial communities associated with the stem xylem of maize plants (Zhang *et al*., 2022a). Members of these communities were isolated and re‐established with the plants, where they were observed to be in the xylem sap via fluorescence microscopy. Through ^15^N isotopic dilution experiments, it was shown that N_2_‐fixing strains were able to provide up to *c*. 12% of the total accumulated N in maize stems. The authors concluded that those bacteria observed in the xylem sap could have been responsible for increased plant N and would represent ‘an untapped resource that can be exploited to increase crop productivity’. With our analytical approach, one could now build on these data and provide further support for *in situ* N_2_‐fixation activity of xylem‐associated diazotrophs. This would allow not only to eradicate doubts whether or not these xylem‐associated diazotrophs are truly active *in situ*, but also to assess their activity across the community to obtain an improved estimation of their contribution to plant nutrition. Similar considerations apply to other studies in the field of root‐associated diazotrophs, ranging from communities associated with roots of maize (Van Deynze *et al*., 2018), rice (Jiang *et al*., 2022; Yan *et al*., 2022), silvergrass (Li *et al*., 2022), and sugarcane (Boddey *et al*., 1995), to marine seagrasses (Martin *et al*., 2020). In addition, investigating plant‐associated diazotrophy through the lens of correlative microscopy and chemical imaging has the potential to inform conceptual models such as mucilage‐assisted N_2_ fixation associated with cereal crops (Bennett *et al*., 2020).

Besides expanding our basic understanding of plant–microbe associations and interactions, we believe that our analytical approach also allows gathering valuable information for management of agricultural systems. Crop production increasingly relies on the application of N‐containing industrial fertilizers (Ladha *et al*., 2016). In rice cultivation, the potential of biological nitrogen fixation is estimated to account for up to 33% of the total N demand of some varieties (Ladha & Reddy, 2003; Chalk, 2016). Although a multitude of strains are commercialized as potential bio‐fertilizers for crops, direct evidence of bacterial N_2_ fixation on and within plant tissues has been missing to date. For rice plants, previous inoculation experiments with isolated strains (e.g. *Azoarcus olearius*) demonstrated the expression of *nif* genes in diazotrophs, which were presumably fixing N_2_ (Egener & Hurek, 1999; Faoro *et al*., 2017; Sarkar *et al*., 2017; Jiang *et al*., 2022). Their actual *in situ* activity, however, has not been confirmed yet, corroborating the ongoing debate on the correlation between plant growth promotion and N_2_ fixation activity of individual bacterial strains (Cassán & Diaz‐Zorita, 2016; Chalk, 2016; Dixon & Hartmann, 2017). Investigating the N_2_ fixation activity of commercial products denoted as N_2_‐fixing bacteria *in situ* could help to substantiate whether and to what extent these strains indeed help to provide N to plants in field situations.

### Incorporating the Gold‐FISH‐NanoSIMS analytical approach in microbial activity investigations

**Fig. 4:**
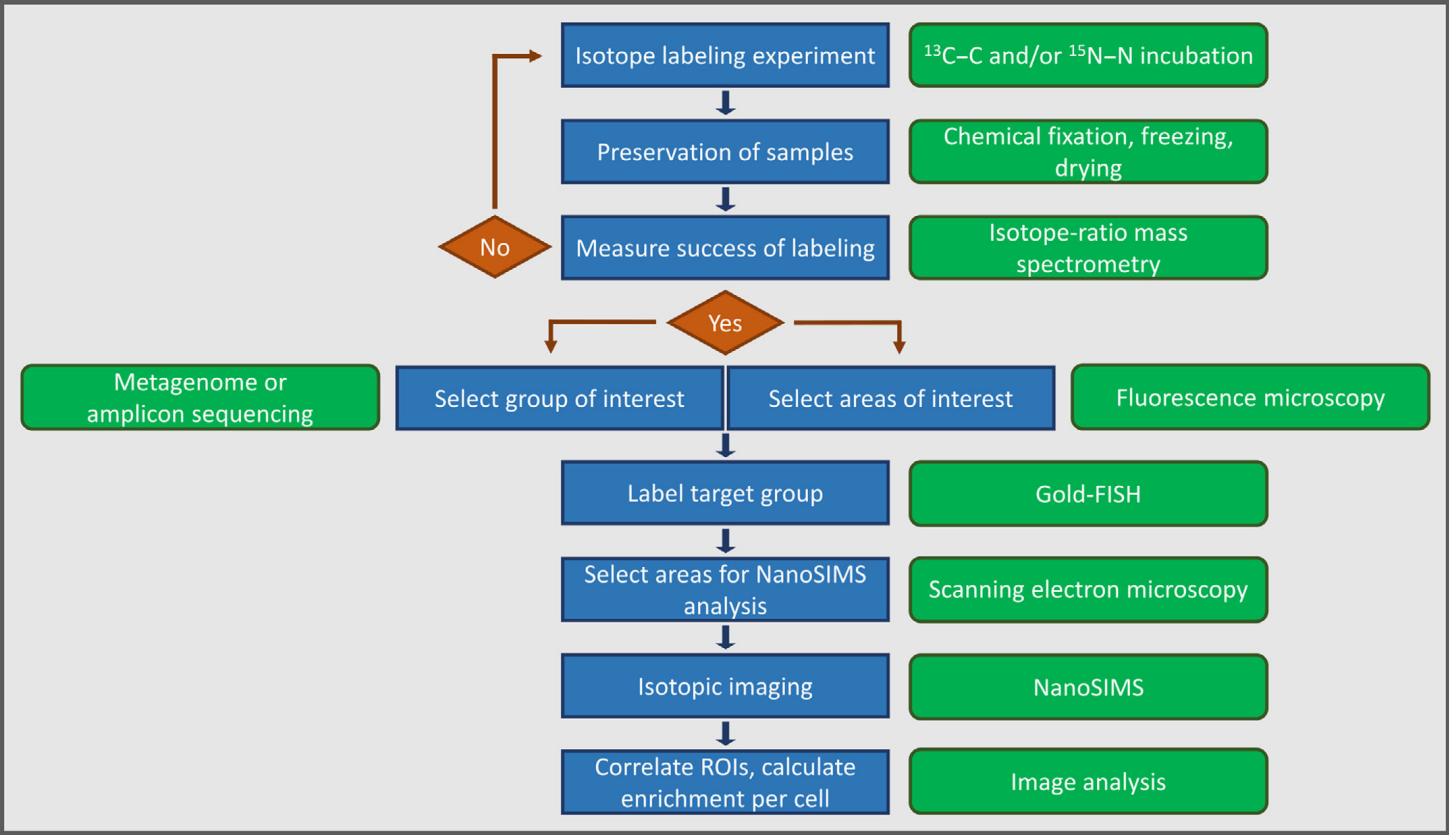
Integrating the analytical approach of Gold‐fluorescence in situ hybridization (FISH)‐nanoscale secondary ion mass spectrometry (NanoSIMS) in an experiment using stable isotope labeling with ^13^C–C and/or ^15^N–N comprising subsequent analysis steps involving mass spectrometry, sequencing, correlative microscopy, and isotopic imaging. Individual experimental steps are described in blue rectangles while green boxes refer to applicable analytical techniques. Blue arrows show the direction of the experimental process. Red arrows indicate to either proceed with the analytical approach (‘Yes’) or repeat the labeling experiment (‘No’) depending on its success as evaluated by mass spectrometry.

Correlative imaging and NanoSIMS analysis are time‐ and cost‐intensive. Consequently, we recommend performing preliminary evaluation to increase the likelihood that the specimen of interest will yield valuable data on the single‐cell scale. Before applying our analytical approach, we suggest determining the isotopic enrichment of replicate bulk samples such as milled roots (Fig. 4). If the highly sensitive measurements through isotope‐ratio mass spectrometry do not indicate enrichment in the targeted stable isotope, we recommend discontinuing with the workflow. Knowledge of the spatial patterns of microbial distribution on or in any given specimen can help to increase the probability of retrieving a sufficiently high number of gold‐labeled, target cells that may have incorporated the isotope of interest. We performed NanoSIMS measurements only within regions of the root exhibiting a high probability of being densely colonized by *Kosakonia* strain DS‐1. Preceding evaluation of colonization patterns via epifluorescence microscopy on replicate samples aided in identification of the regions with increased bacterial colonization (Schmidt *et al*., 2018). If environmental samples were to be analyzed without *a priori* knowledge on the target groups of interest, accompanying sequencing of marker genes or metagenomes will be valuable to select taxa of interest to be detected via Gold‐FISH.

Following these directions and the workflow presented in Fig. 4, future research could assess whether (1) individual or groups of taxa identified by sequencing (e.g. amplicon sequencing of marker genes or metagenomics) are indeed participating in a process of interest or if (2) bio‐inoculants such as plant growth‐promoting bacteria and/or engineered strains effectively interact with their plant host by performing the requested function. The presented approach could be expanded beyond ^15^N_2_ incubations by using ^13^C‐labeled compounds (e.g. ^13^C–CO_2_). This would allow testing for the uptake of root exudates into bacterial cells along the rhizoplane and thus also include spatio‐temporal aspects of plant–microbe interactions and nutrient cycling in the rhizosphere. It is important to note here that labeling of root‐associated microbiota through exudates may require extended incubation time. In addition, coating with a thin layer of carbon (*c*. 20 nm) used to prevent electrical charging during SEM and NanoSIMS analysis could result in a dilution of the ^13^C signal in microbial cells. We recommend testing potential dilution effects on ^13^C‐labeled microbial cultures with and without C coating before the plant‐labeling experiment. This would allow to estimate the dilution effect of the C coating and to make a data‐informed decision on the chances for success of such an experiment. Another interesting application could be the analysis of multi‐kingdom interactions in the plant–fungal–microbe interface. The so‐called ‘hyphosphere’ is currently evolving into a major field of research while still being rather understudied with regard to the role bacteria play in plant–fungal interactions (Gorka *et al*., 2019; See *et al*., 2022; Zhang *et al*., 2022b). In general, we believe that our approach can be useful to provide evidence‐based clarification of current uncertainties regarding *in situ* microbial activity and plant–microbe interactions.

## Competing interests

None declared.

## Author contributions

HS and DW established the idea and designed the experiment in consultation with DS. HS and DS performed gnotobiotic experiments and all sample preprocessing including microscopy and spot selection in consultation with AS. AS conducted the NanoSIMS analysis in consultation with HS. SG and HS analyzed the NanoSIMS data and prepared figures in consultation with AS and DW. HS wrote the manuscript with contributions from all co‐authors.

## Supporting information


**Fig. S1** Delay between the detection of gold and ^15^N enrichment in cellular biomass.
**Fig. S2** Selection of regions of interest.
**Fig. S3** NanoSIMS determination of the ^15^N label dilution effect of Gold‐FISH.
**Fig. S4** Effect of the Cs^+^ primary ion fluence on the correlation of gold‐labeled bacteria with areas of ^15^N enrichment on the root surface.
**Fig. S5** Gold‐FISH stained bacteria on rice roots as imaged via SEM‐SE or SEM‐BSE.
**Fig. S6** Effect of regions of interest selection on the evaluated ^15^N content.
**Fig. S7** Specificity of Gold‐FISH labeling.
**Methods S1** Gold‐FISH of root‐associated *Kosakonia* strain DS‐1 cells.
**Methods S2** Preparation and microscopic characterization of samples before NanoSIMS analysis.
**Notes S1** Specificity of Gold‐FISH for identification of target bacteria.
**Notes S2** Effects of contamination of ^15^N–N_2_ with ^15^N–NH_3_ in this study.

**Table S1** Contamination assessment of ^15^N–N_2_ gas used for gnotobiotic experiment.

**Video S1** Region of interests and ^15^N enrichment in all 95 acquisition cycles of Analysis Area 1.
Please note: Wiley is not responsible for the content or functionality of any Supporting Information supplied by the authors. Any queries (other than missing material) should be directed to the *New Phytologist* Central Office.

## Data Availability

The data that support the findings of this study are openly available on Zenodo at https://doi.org/10.5281/zenodo.8043397 (Schmidt *et al*., 2023).
