# Supplementary material for: Gold‐FISH enables targeted NanoSIMS analysis of plant‐associated bacteria
Source: New Phytol. 2023 Jun 28;240(1):439–51. doi: 10.1111/nph.19112 (PMC10962543; doi:10.1111/nph.19112)
Supplement: Supplementary file 1 — Fig. S1 Delay between the detection of gold and 15N enrichment in cellular biomass. Fig. S2 Selection of regions of interest. Fig. S3 NanoSIMS determination of the 15N label dilution effect of Gold‐FISH. Fig. S4 Effect of the Cs+ primary ion fluence on the correlation of gold‐labeled bacteria with areas of 15N enrichment on the root surface. Fig. S5 Gold‐FISH stained bacteria on rice roots as imaged via SEM‐SE or SEM‐BSE. Fig. S6 Effect of regions of interest selection on the evaluated 15N content. Fig. S7 Specificity of Gold‐FISH labeling. Methods S1 Gold‐FISH of root‐associated Kosakonia strain DS‐1 cells. Methods S2 Preparation and microscopic characterization of samples before NanoSIMS analysis. Notes S1 Specificity of Gold‐FISH for identification of target bacteria. Notes S2 Effects of contamination of 15N–N2 with 15N–NH3 in this study. [file NPH-240-439-s002.pdf]

New Phytologist **Supporting Information**

Article title: Gold-FISH enables targeted NanoSIMS analysis of plant-associated bacteria
Authors: Hannes Schmidt, Stefan Gorka, David Seki, Arno Schintlmeister, Dagmar Woebken
Article acceptance date: 14 June 2023

The following Supporting Information is available for this article:

Methods S1 Gold-FISH of root-associated Kosakonia strain DS-1 cells

Table S1 Contamination assessment of ^{15}N-N_2 gas used for gnotobiotic experiment (available as separate Excel file)

Methods S2 Preparation and microscopic characterization of samples prior to NanoSIMS analysis

Fig. S1 Delay between the detection of gold (panel a) and ^{15}N enrichment in cellular biomass

Fig. S2 Selection of regions of interest (ROIs)

Fig. S3 NanoSIMS determination of the ^{15}N label dilution effect of Gold-FISH

Fig. S4 Effect of the Cs^+ primary ion fluence on the correlation of gold-labeled bacteria with areas of ^{15}N enrichment on the root surface

Fig. S5 Gold-FISH stained bacteria on rice roots as imaged via SEM-SE or SEM-BSE

Fig. S6 The effect of ROI selection on the evaluated ^{15}N content

Fig. S7 Specificity of Gold-FISH labeling

Notes S1 Specificity of Gold-FISH for identification of target bacteria

Notes S2 The effects of contamination of ^{15}N-N_2 with ^{15}N-NH_3 in this study

Video S1 ROIs and ^{15}N enrichment in all 95 acquisition cycles of Analysis Area 1 (available as separate gif file)

Methods S1 Roots were cut into segments of approximately 0.5 cm in length. Root segments representing the elongation zone were used for Gold-FISH as these areas exhibited a dense

colonization by Kosakonia strain DS-1 in parallel experiments (Schmidt et al., 2018). Up to five root sections were incubated in 400 µL hybridization buffer (0.9 M NaCl, 20 mM Tris–HCl, 10% blocking reagent (Roche, Germany), 0.01% SDS, 10 % dextran sulfate (Sigma-Aldrich, Germany), 35% formamide (Carl Roth, Germany) containing 50 ng µL-1 of horseradish peroxidase labeled oligonucleotide probes (EUBI-III, Daims et al., 1999; Biomers.net, Germany) for 3 h at 46 °C. Afterwards, the root sections were consecutively washed in pre-warmed washing buffer (70 mM NaCl, 20 mM Tris, 5 mM EDTA, 0.01% SDS; 5 min at 48 °C), H2OMQ (2 min at RT), and 0.05% Triton-X100 (Bio-Rad, Germany) in 1 × PBS for 5 min at RT. For CARD, the hybridized root segments were incubated in 450 µL of amplification buffer (1 × PBS containing 10% dextran sulfate, 1 % blocking reagent, 2 M NaCl) amended with 0.0015% H2O2 and 20 mg mL-1 biotinylated tyramide solution (in dimethylformamide, prepared according to Hopman et al., 1998) containing 2 % 3-iodophenolboronic acid (Sigma-Aldrich, Germany) for 20 min at 46 °C. Root segments were washed in 1 × PBS containing 0.1 % gelatine (PGT), 0.1 % Tween-20 (Sigma-Aldrich, Germany) and H2OMQ for 10 min at RT, respectively. Root segments were incubated in 400 µL 1 × PBS containing 1% BSA (fraction V without biotin, Carl Roth, Germany) and 0.08 mg mL-1 of AlexaFluor®488FluoroNanogoldTM-Streptavidin (Nanoprobes, USA) for 5 h at 46°C. Afterwards, root segments were washed in PGT and H2OMQ for 5 min at RT, respectively. Following three washes in NaCl-Tween (1M NaCl, 0.5% Tween-20) and H2OMQ for 5 min each, the root segments were transferred into a gold developer solution (GoldEnhanceTM EM Plus, Nanoprobes, USA) for auto-metallographic deposition of gold on the gold-nanoparticles introduced by Gold-FISH, thus enhancing grain size and facilitating detection via SEM and NanoSIMS. Therefore, 1 part enhancer solution was mixed with 3 parts of activator solution and pre-incubated for 4 minutes. Afterwards, 1 part of initiator and buffer solutions were added, respectively. Root segments were immersed in the gold developer solution and incubated for 10 min at RT. Consecutive washing steps in H2OMQ amended with 1% Na2S2O3 and H2OMQ (twice) were performed for 5 min at RT, respectively. Root segments were either stored in 1 x PBS:EtOH (2:3, vol:vol) at -20 °C or immediately used for mounting as described in the main text, section ‘Preparation and microscopic characterization of samples prior to NanoSIMS analysis’.

Table S1 Contamination assessment of ^{15}N-N_2 gas used for gnotobiotic experiment.

Methods S2 All samples were deposited on antimony-doped silicon wafer platelets (7.1 × 7.1 × 0.75 mm, Active Business Company, Germany) pre-coated with Vectabond (Vector Laboratories Inc., USA) to improve adhesion of the filamentous root segments to the wafer surface. Suspensions of fixed pure cultures of Kosakonia strain DS-1 were diluted in H2OMQ and each 5 µL were spotted onto individual wafers. Wafers were dried in an incubator at 46°C and either processed via Gold-FISH including DAPI staining (1 µg/mL DAPI in 1 x PBS, 10 min, RT) or immediately subjected to laser accomplished marking and stereo microscopy as described below. Mounting of roots was performed exclusively by deposition on the wafer surface, without any resin embedding or gluing. Small lateral roots were removed from individual root segments under a stereoscope and discarded. Root segments of approximately 0.5 cm in length were then placed in a droplet of H2OMQ on a silicon wafer platelet and air-dried. Afterwards, the specimens were coated with a thin film of carbon of approximately 20 nm thickness (EM MED20, Leica, Germany) to prevent electrical charging during SEM and NanoSIMS analysis.A confocal laser scanning microscope (TCS SP8X, Leica, Germany), equipped with a 63x glycerol objective, was used to verify successful Gold-FISH hybridization on replicate pure cultures and replicate root segments (mounted onto glass slides). DAPI was excited by a 405 nm diode laser and detected at a wavelength of 450 nm. Alexa488 was excited using a white-light laser at 488 nm and detected in the spectral bandwidth between 510-550 nm. In order to select appropriate analysis areas for NanoSIMS, the silicon wafer platelets with the spotted pure cultures were imaged and marked using a laser microdissection microscope (DM6000 B equipped with LMD 7000, Leica, Germany) with the following settings: magnification: 63×, power: 10, aperture: 1, speed: 60, specimen balance: 27, head current: 70%, pulse frequency: 201. Silicon wafer platelets with air-dried root segments were imaged by a scanning electron microscope (SEM; JEOL IT 300, Germany) to screen root surfaces for microbial colonization through the detection of gold signals. Regions that showed bacterial colonization, as visualized through gold deposition, were then selected as potential analysis areas for NanoSIMS based on the observed

bacterial cell density and root topography. Secondary electron (SE) and backscattered electron (BSE) images were acquired by the respective detectors at various magnifications and a working distance of 9-12 mm, High P.C. 40-50, and 15-20 kV. The presence of gold in and on microbial cells was verified via energy dispersive X-ray spectroscopy (Ametek, Germany) for selected samples. A stereo microscope was further used to determine the relative position of the roots on the wafer platelets, which facilitated localization of the SEM pre-selected analysis areas for NanoSIMS, guided by the video images from the CCD camera installed on the NanoSIMS instrument.

Fig. S1 Delay between the detection of gold (panel a) and ^{15}N enrichment in cellular biomass (panel b) as observed on Gold-FISH treated single cells from Kosakonia strain DS-1 (c.f. Fig. S3, panel b). Each cycle corresponds to Cs^+ ion irradiation with a fluence of 2.4E15 ions/cm^2. Note that the $^{197}Au^-$ signal intensity has been normalized to $^{12}C_2^-$ since $^{12}C^-$ secondary ions were not detected in this particular measurement. However, the flat arrangement of the cells on the wafer surface and the confinement of ROIs to the lumen of the cells restricted the influence of topography, which is also resembled by the raw signal intensity of $^{197}Au^-$ showing exactly the same trend (inset in panel b). Solid lines display the mean value over 91 individual ROIs, the shaded areas refer to values within +/- one standard deviation.

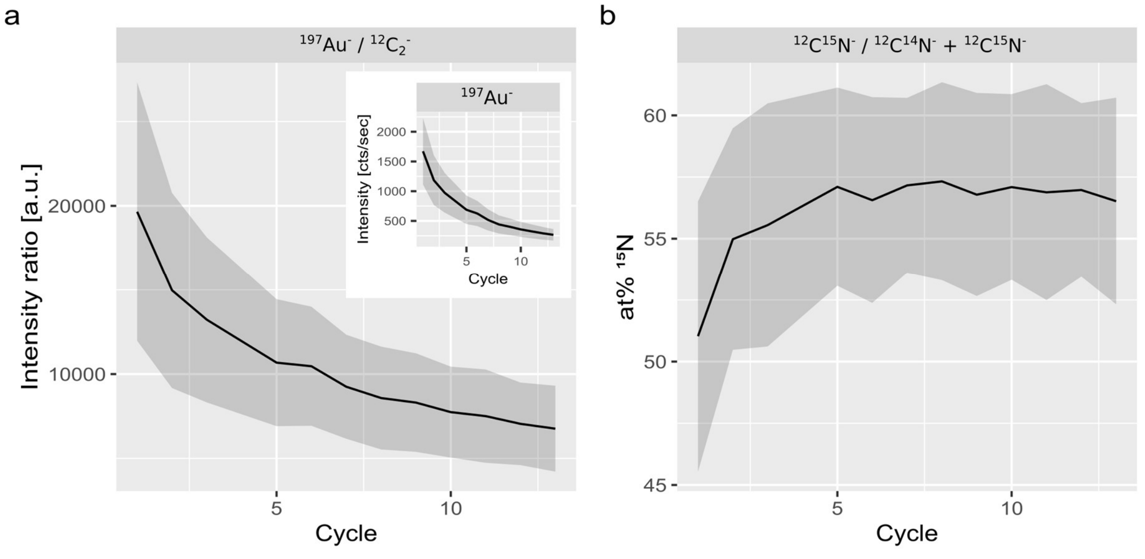

Fig. S2 Selection of regions of interest (ROIs) as indicated in images from SEM-BSE (panel a), NanoSIMS-$^{197}Au^-$ secondary ion (panel b) and NanoSIMS-secondary electron detection (panel c). ROIs based selectively on the presence of only gold are shown as blue circular shapes, ROIs associated with the presence of only gold and phosphorus as green circular shapes and ROIs referring to the presence of gold and phosphorus in combination with ^{15}N isotope enrichment as red circular shapes. Signal intensities are displayed on a grey-scale, ranging from black (low) to white (high). Scale bars: 5 µm.

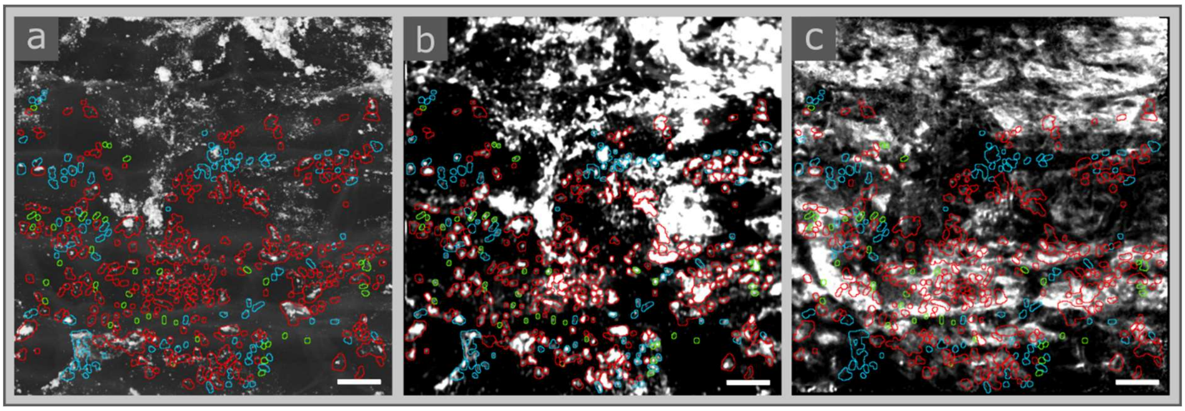

Fig. S3 Pure cultures of *Kosakonia* strain DS-1 were grown in semisolid liquid medium either without any nitrogen source (natural abundance control) or amended with ^{15}N-NH_4Cl. A fraction of the latter was further subjected to the Gold-FISH procedure in order to assess the potential decrease of the ^{15}N content commonly observed for FISH-staining in NanoSIMS studies (Musat *et al.*, 2014; Woebken *et al.*, 2015b; Musat *et al.*, 2016; Stryhanyuk *et al.*, 2018). Single cells were spotted onto wafers and analyzed regarding their ^{15}N enrichment (Figure S5). Cells grown in the presence of ^{15}N-NH_4Cl and not subjected to Gold-FISH showed a tracer content of 94.7 +/- 0.9 at% ^{15}N (mean, SD). Cells from the identical culture but treated with Gold-FISH showed a mean enrichment of 56.0 at% ^{15}N corresponding to a nitrogen dilution factor (DF), as defined in (Woebken *et al.*, 2015a; Meyer *et al.*, 2021), of 1.64 originating from loss of cellular biomass through permeabilization and the deposition of compounds with natural isotopic abundance.

NanoSIMS determination of the ^{15}N label dilution effect of Gold-FISH on single cells from Kosakonia strain DS-1. Pure culture of Kosakonia strain DS-1 grown on ^{15}N-NH_4 before (panel a) and after (panel b) Gold-FISH treatment. at% ^{15}N values are displayed on a false-color scale ranging from 0.63 (dark blue) to 70 (red). Black areas refer to pixels in which an unbiased determination of the ^{15}N content was not feasible due to low counting statistics (silicon wafer surface). Panel c shows the results obtained from single cell-specific data evaluation (n = 78 and 91 for Gold-FISH untreated and treated cells, respectively). Boxplots illustrate summary statistics, comprising the median (=line), first and third quartile (=box), and 1.5x the interquartile range (=whiskers). Scale bars: 5 µm.

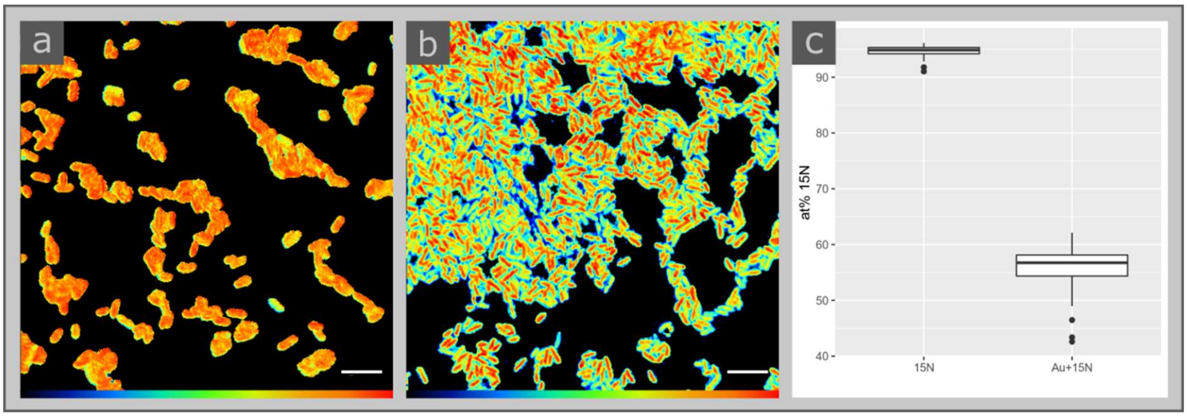

Fig. S4 Effect of the Cs^+ primary ion fluence on the correlation of gold-labeled bacteria with areas of ^{15}N enrichment on the root surface. Gold particles appear as bright spots in SEM-BSE images (left column) and as hot-spots (yellow-red) in NanoSIMS-$^{197}Au^-$ secondary ion maps (central column). Colored areas in the NanoSIMS at% ^{15}N images (right column) refer to regions of local ^{15}N enrichment (i.e. at% $^{15}N > 0.63$, see Material and Methods). Five areas of AA1 are highlighted (panels A-E). The effect of the delay in the NanoSIMS Au and ^{15}N label detection is exemplarily illustrated by images recorded in the acquisition cycles 5, 30, and 80, corresponding to a Cs^+ fluence of 1.1E16, 5.6E16 and 1.4E17 ions/cm^2, respectively. Scale bars: top: 5 µm; bottom: 1 µm

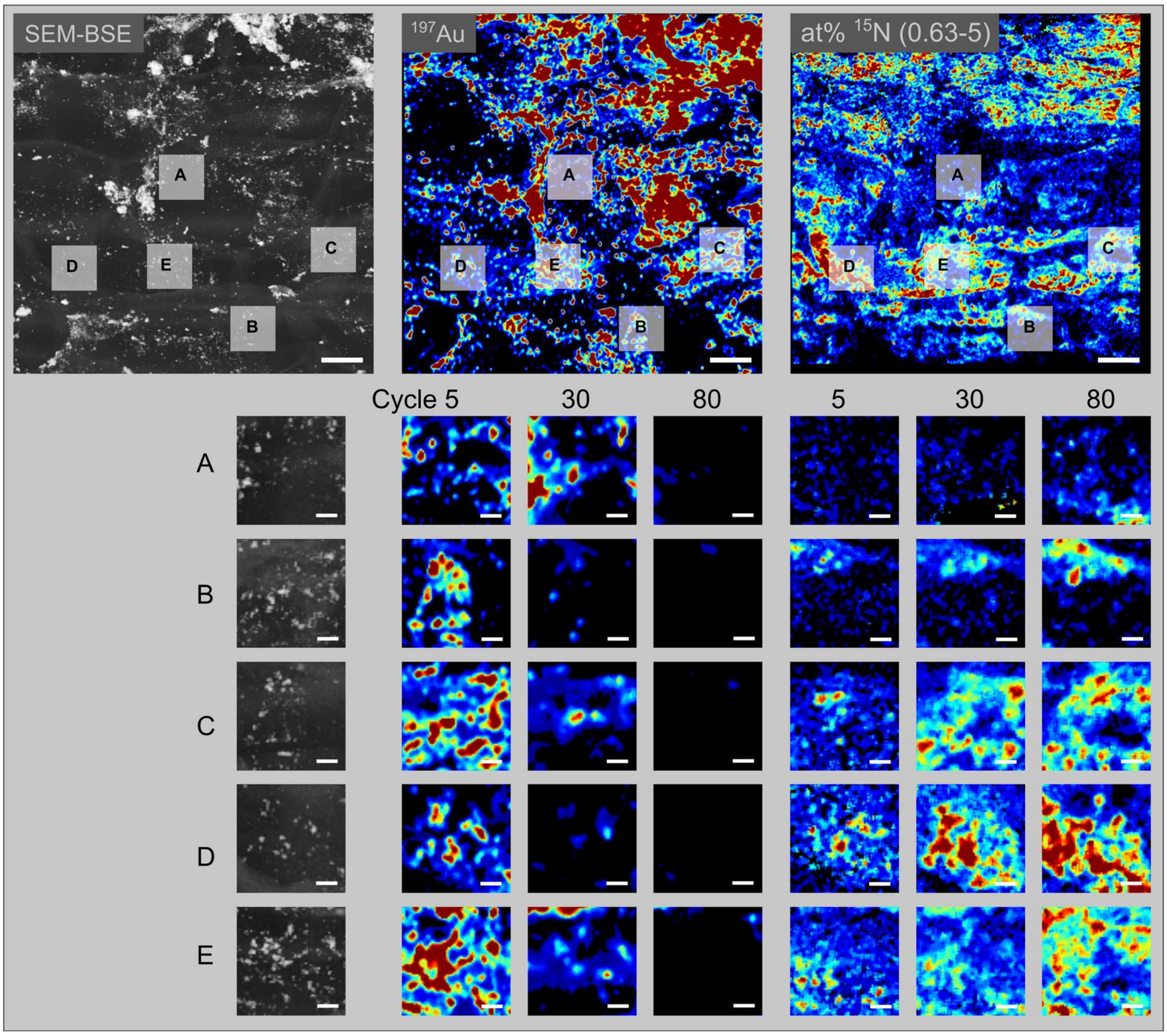

Fig. S5 Gold-FISH stained cells of Kosakonia strain DS-1 on the surface of gnotobiotically grown rice roots as imaged via SEM-SE (panel a) or SEM-BSE (panel b). Individual bacteria are less recognizable in the SEM-SE image as compared to the SEM-BSE acquisition where the gold labeling appears as bright spots. Scale bars: 20 µm.

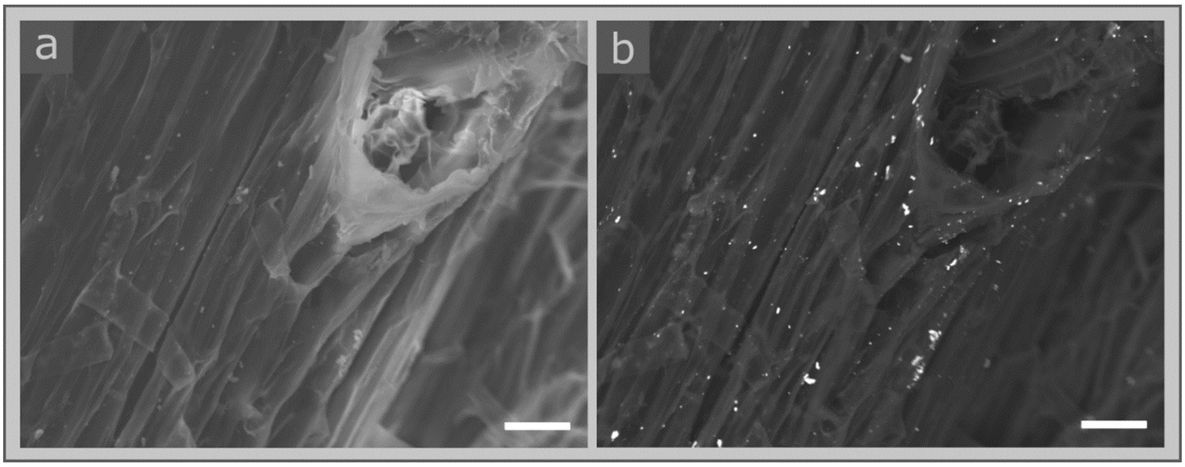

Fig. S6 Boxplot illustrating the effect of ROI selection on the evaluated ^{15}N content. Values refer to the data obtained from selected ROIs over 95 image acquisition cycles in Analysis Area 1.

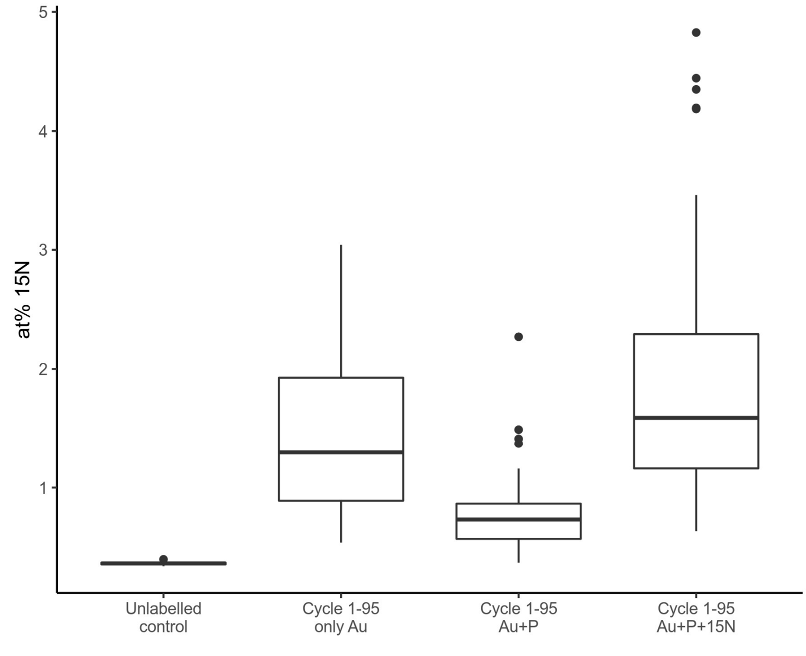

Fig. S7 Gold-FISH specifically labels cells of B. subtilis (target organism) mixed with a culture of Kosakonia strain DS-1 (non-target organisms). Panel a: Fluorescence micrograph highlighting the presence of both bacterial strains as stained by DAPI. Panel b: SEM-BSE shows specific labeling of B. subtilis (white rectangle) and no unspecific labeling of Kosakonia strain DS-1 (yellow arrow and rectangle). Panel c: Magnified area of panel b. Panel d: Energy-dispersive X-ray spectroscopy (EDX) confirms the presence of Au in cells of B. subtilis specifically labeled via Gold-FISH. Scale bars: 20 µm.

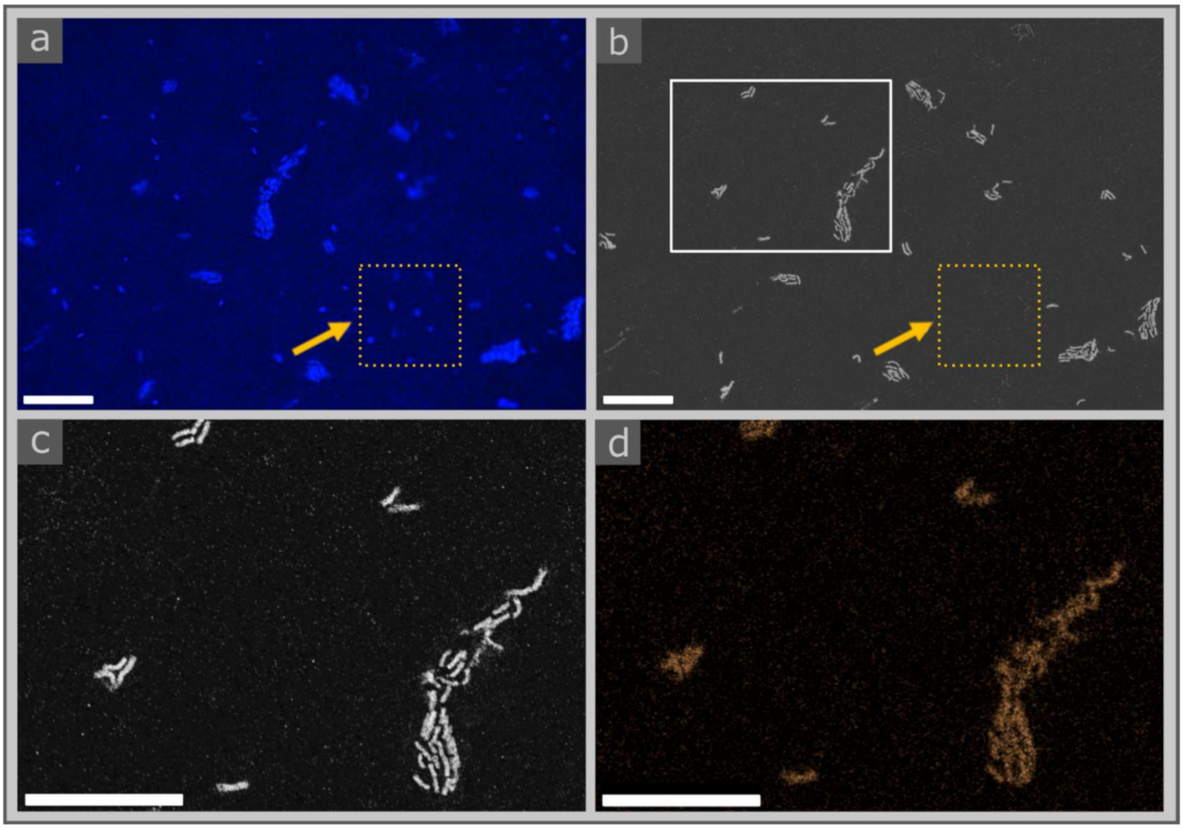

Notes S1 Specificity of Gold-FISH for identification of target bacteria. An important feature of Gold-FISH is that the deposition of gold nanoparticles and their autometallographic enhancement are specific to the target cells without yielding a high number of false positives. In addition to existing evidence of its specificity (Schmidt et al., 2012; Eickhorst and Schmidt, 2015), we applied Gold-FISH to a mixed culture of *Kosakonia* strain DS-1 and *Bacillus subtilis* (DSMZ 10). *Kosakonia* strain DS-1 was grown as described in the main text (see 'Cultivation of Kosakonia strain DS-1'). *B. subtilis* was grown in 25 ml liquid LB medium (Bertani, 1951) at ambient oxygen levels and room temperature. After 12 h of growth, *B. subtilis* cells were pelleted by centrifugation, washed twice in 1 × PBS, and centrifuged again. For fixation, the resulting pellet was re-suspended in molecular grade EtOH for 1 hour at room temperature and stored in 1 x PBS/EtOH (vol:vol) at -20°C. Fixed cells of *B. subtilis* and *Kosakonia* strain DS-1 were transferred to a polycarbonate filter (0.2 µm pore size; Millipore). Hybridization was performed as described in the main text using the probe LGC354A (Meier et al., 1999) specific for *Firmicutes* (i.e. *B. subtilis*) but not for *Gammaproteobacteria* (i.e. *Kosakonia* strain DS-1). Scanning electron microscopy revealed specific staining of *B. subtilis* cells via Gold-FISH as compared to the DAPI-stain (visualized via fluorescence imaging) that also stained cells of *Kosakonia* strain DS-1 (Figure S2). Afterwards, we applied autometallographic enhancement of gold nanoparticles and observed the same *B. subtilis* cells to be gold-stained as well while no false positives were observed for *Kosakonia* strain DS-1. Subsequent energy-dispersive X-ray spectroscopy (EDX) verified the co-occurrence of gold associated with our target cells.

Notes S2 The effects of contamination of $^{15}N\text{-}N_2$ with $^{15}N\text{-}NH_3$ in this study. After 72 hours of incubation with $^{15}N\text{-}N_2$ in a closed and otherwise N-free gnotobiotic system, most of the ^{15}N was found in the aboveground plant material (see Supporting Information Table S1). This suggested that ^{15}N-ammonia gas was mainly absorbed by the leaves due to their comparatively high surface area (Hutchinson et al., 1972; Artyomov et al., 1994). If ^{15}N-ammonia indeed dissolved into the aqueous solution (pH <8) overlaying the growing medium for roots, we are convinced that the high ammonium uptake rates previously reported for rice plants of a similar

age (Wang et al., 1993; Kronzucker et al., 1998) would have depleted the ^{15}N-ammonium available for cells of *Kosakonia* strain DS-1 in this otherwise ^{15}N-free experimental system. Therefore, we are convinced that most of the ^{15}N-ammonia present after injection of the ^{15}N gas was immediately immobilized by the plant. If ^{15}N-ammonium was still available in the water film around rice roots (e.g. through root loss), we assume it would have been taken up homogeneously among individual bacterial cells along the rhizoplane, something we did not observe in our study. We found that approximately 66% of gold-labeled cells detected on the root surface also showed ^{15}N enrichment, indicating commonly observed heterogeneous activity patterns among members of clonal bacterial populations (Volland et al., 2018; Calabrese et al., 2019).

Video S1 ROIs and ^{15}N enrichment in all 95 acquisition cycles of Analysis Area 1. at% ^{15}N values are displayed on a false-color scale ranging from 0.63 (dark blue) to 4 (red). Acquisition cycles are shown on the left corner as numerals. White circular shapes show the ROIs selected for NanoSIMS analysis. Scale bar: 5µm.

Supporting References

Artyomov VM, Artyomov EM, Fridman SD. **1994**. Ammonia uptake by plants. *Environmental Monitoring and Assessment* **29**: 221–228.

Bertani G. **1951**. Studies on lysogenesis. I. The mode of phage liberation by lysogenic Escherichia coli. *Journal of Bacteriology* **62**: 293–300.

Calabrese F, Voloshynovska I, Musat F, Thullner M, Schlömann M, Richnow HH, Lambrecht J, Müller S, Wick LY, Musat N, *et al.* 2019. Quantitation and Comparison of Phenotypic Heterogeneity Among Single Cells of Monoclonal Microbial Populations. *Frontiers in Microbiology* **10**: 1–23.

Hutchinson GL, Millington RJ, Peters DB. **1972**. Atmospheric Ammonia: Absorption by Plant Leaves. *Science* **175**: 771–772.

Kronzucker HJ, Kirk GJD, Siddiqi MY, Glass ADM. **1998**. Effects of Hypoxia on 13NH4+ Fluxes in Rice Roots: Kinetics and Compartmental Analysis. *Plant Physiology* **116**: 581–587.
Meier H, Amann R, Ludwig W, Schleifer KH. **1999**. Specific oligonucleotide probes for in situ detection of a major group of gram-positive bacteria with low DNA G+C content. *Systematic and Applied Microbiology* **22**: 186–196.
Meyer NR, Fortney JL, Dekas AE. **2021**. NanoSIMS sample preparation decreases isotope enrichment: magnitude, variability and implications for single-cell rates of microbial activity. *Environmental Microbiology* **23**: 81–98.
Musat N, Musat F, Weber PK, Pett-Ridge J. **2016**. Tracking microbial interactions with NanoSIMS. *Current Opinion in Biotechnology* **41**: 114–121.
Musat N, Stryhanyuk H, Bombach P, Adrian L, Audinot JN, Richnow HH. **2014**. The effect of FISH and CARD-FISH on the isotopic composition of 13C- and 15N-labeled Pseudomonas putida cells measured by nanoSIMS. *Systematic and Applied Microbiology* **37**: 267–276.
Stryhanyuk H, Calabrese F, Kümmel S, Musat F, Richnow HH, Musat N. **2018**. Calculation of single cell assimilation rates from sip-nanosims-derived isotope ratios: A comprehensive approach. *Frontiers in Microbiology* **9**: 1–15.
Volland JM, Schintlmeister A, Zambalos H, Reipert S, Mozetič P, Espada-Hinojosa S, Turk V, Wagner M, Bright M. **2018**. NanoSIMS and tissue autoradiography reveal symbiont carbon fixation and organic carbon transfer to giant ciliate host. *ISME Journal* **12**: 714–727.
Wang MY, Siddiqi MY, Ruth TJ, Glass ADM. **1993**. Ammonium uptake by rice roots. *Plant Physiology* **103**: 1249–1258.
Woebken D, Burow LC, Behnam F, Mayali X, Schintlmeister A, Fleming ED, Prufert-Bebout L, Singer SW, Cortés AL, Hoehler TM, *et al.* **2015a**. Revisiting N2 fixation in Guerrero Negro intertidal microbial mats with a functional single-cell approach. *ISME Journal* **9**: 485–496.
Woebken D, Burow LC, Behnam F, Mayali X, Schintlmeister A, Fleming ED, Prufert-bebout L, Singer SW, Ló Pez Cortés A, Hoehler TM, *et al.* **2015b**. Revisiting N 2 fixation in Guerrero Negro intertidal microbial mats with a functional single-cell approach. *The ISME journal* **9**: 485–496.
