# Supplementary figures and images for: Gold‐FISH enables targeted NanoSIMS analysis of plant‐associated bacteria
Source: New Phytol. 2023 Jun 28;240(1):439–51. doi: 10.1111/nph.19112 (PMC10962543; doi:10.1111/nph.19112)

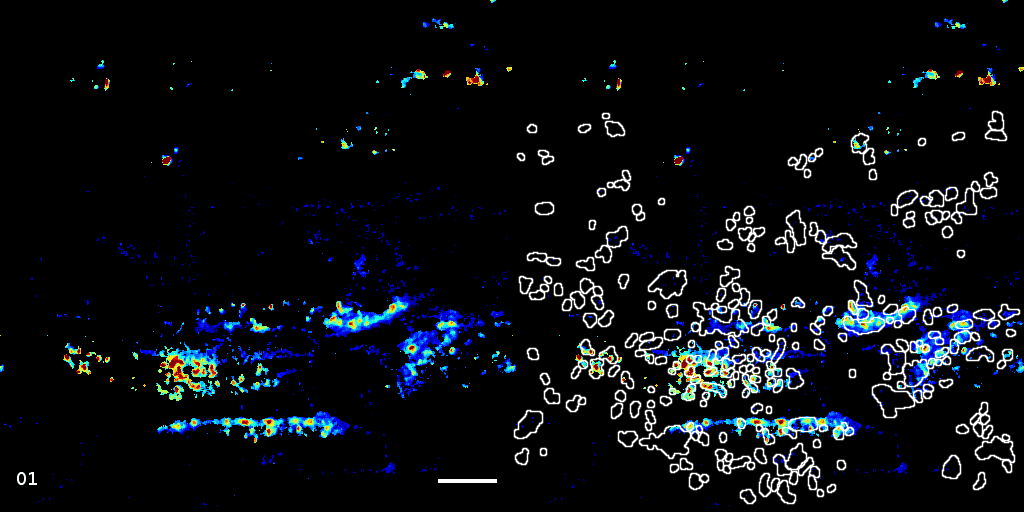

Supplement: Supplementary file 3 — Video S1 Region of interests and 15N enrichment in all 95 acquisition cycles of Analysis Area 1. Please note: Wiley is not responsible for the content or functionality of any Supporting Information supplied by the authors. Any queries (other than missing material) should be directed to the New Phytologist Central Office. [file NPH-240-439-s001.gif]
